# Supplementary material for: Baseline Characterization of the Gut Microbiota of Field and Colony Populations of Phlebotomus tobbi and Preliminary Assessment of the Anti-Leishmanial Activity of Cultivable Bacteria
Source: Pathogens. 2026 Jun 23;15(7):658. doi: 10.3390/pathogens15070658 (PMC13416292; doi:10.3390/pathogens15070658)
Supplement: Supplementary file 1 [file pathogens-15-00658-s001.zip › pathogens-4357168-File S1/Taxa2ASV_Decomposer/Genus/g__Serratia/heatmap_with_clustering.pdf]

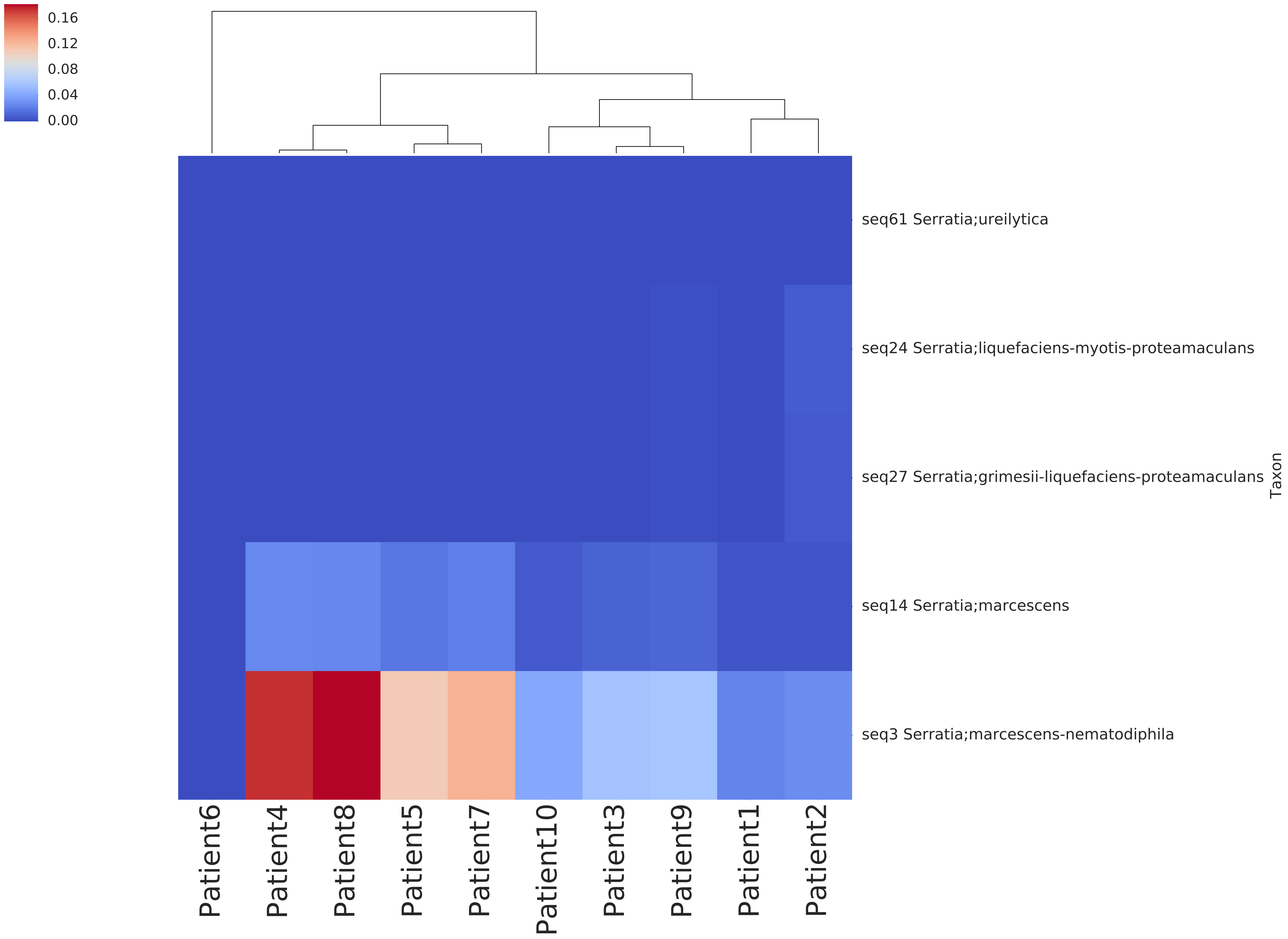
0.16
0.12
0.08
0.04
0.00
seq61 Serratia;ureilytica
seq24 Serratia;liquefaciens-myotis-proteamaculans
seq27 Serratia;grimesii-liquefaciens-proteamaculans
seq14 Serratia;marcescens
seq3 Serratia;marcescens-nematodiphila
Taxon
Patient6
Patient4
Patient8
Patient5
Patient7
Patient10
Patient3
Patient9
Patient1
Patient2
